# Isolation of Antagonistic Endophytes from Banana Roots against *Meloidogyne javanica* and Their Effects on Soil Nematode Community

**DOI:** 10.3389/fmicb.2017.02070

**Published:** 2017-10-26

**Authors:** Lanxi Su, Zongzhuan Shen, Yunze Ruan, Chengyuan Tao, Yifan Chao, Rong Li, Qirong Shen

**Affiliations:** ^1^Jiangsu Provincial Key Lab for Organic Solid Waste Utilization, National Engineering Research Center for Organic-based Fertilizers, Jiangsu Collaborative Innovation Center for Solid Organic Waste Resource Utilization, Nanjing Agricultural University, Nanjing, China; ^2^Spice and Beverage Research Institute, Chinese Academy of Tropical Agricultural Science, Hainan, China; ^3^Hainan Key Laboratory for Sustainable Utilization of Tropical Bio-Resources, College of Agriculture, Hainan University, Hainan, China

**Keywords:** endophytes, antagonism, plant-parasites, *Meloidogyne*, nematode community

## Abstract

Banana production is seriously hindered by *Meloidogyne* spp. all over the world. Endophytes are ideal candidates compared to pesticides as an environmentally benign agent. In the present study, endophytes isolated from banana roots infected by *Meloidogyne* spp. with different disease levels were tested *in vitro*, and in sterile and nature banana monoculture soils against *Meloidogyne javanica*. The proportion of antagonistic endophytes were higher in the roots of middle and high disease levels. Among those, bacteria were dominant, and *Pseudomonas* spp., *Bacillus* spp. and *Streptomyces* spp. showed more abundant populations. One strain, named as SA, with definite root inner-colonization ability was isolated and identified as *Streptomyces* sp. This strain showed an inhibiting rate of >50% *in vitro* and biocontrol efficiency of 70.7% in sterile soil against *Meloidogyne javanica*, compared to the control. Greenhouse experiment results showed that the strain SA exhibits excellent biological control ability for plant-parasites both in roots and in root-knot nematode infested soil. SA treatment showed a higher number of bacterivores, especially *Mesorhabditis* and *Cephalobus*. The maturity index was significantly lower, while enrichment index (EI) was significantly higher in the SA treatment. In conclusion, this study presents an important potential application of the endophytic strain *Streptomyces* sp. for the control of plant-parasitic nematodes, especially *Meloidogyne javanica*, and presents the effects on the associated variation of the nematode community.

## Introduction

The banana is the most popular tropical fruit and consumed widely all over the world (Mendoza and Sikora, 2009). It is a staple food and a source of family income in many countries (Wang et al., 2013). However, its production is hindered by many diseases and pests, including various species of plant-parasitic nematodes, which infest banana roots with adverse consequences (Mendoza and Sikora, 2009). To date, numerous nematode species recognized to cause the most serious damage to banana are *Pratylenchus goodeyi*, *Radopholus similis*, *P. coffeae*, *Helicotylenchus multicinctus*, and *Meloidogyne* spp. (Quénéhervé, 2009). In general, most of banana varieties are the host of root-knot nematodes, which are most likely to be observed in great numbers on banana roots in tropical and subtropical conditions (Quénéhervé, 2009). In sandy soil in southern China, the banana root-knot nematodes *M. javanica* and *M. arenaria* generally occur abundantly, as well as *Rotylenchulus reniformis* and *Helicotylenchus* spp. (Xu et al., 2004). The root-knot nematodes (*Meloidogyne* spp.) can cause the banana yield reduction for 20–30% commonly (Liu et al., 2005). These nematodes not only have short biological cycle, but also cause wounds in the host roots which help in the invasion of secondary pathogens (Caboni et al., 2016).

In the past, nematode control mainly relied on the use of nematicides (Caboni et al., 2016). Reduction of the use of pesticides to protect human health and the environment has increasingly caused public concern. Compared to other effective management measures, biocontrol with advantages related to longevity, safety, environmental conservation is considered to be a promising strategy (Wang et al., 2013). Thus, exploring naturally generating plant-associated microorganisms with beneficial effects to plant health offers an unprecedented opportunity for plant protection (Compant et al., 2005). At present, most commercial biocontrol products on the market contain live microorganisms, such as *Bacillus firmus*, *Pasteuria penetrans*, *Purpureocillium lilacinus*, and/or their metabolites, which target specific nematodes (Lamovšek et al., 2013). Of particular interest are actinomycetes (Oka et al., 2000), some of which produce metabolic substances to inhibit or even kill nematodes, as well as trigger plant induced systemic resistance (ISR) (Qin et al., 2011). Multiple commercial products based on actinomycetes have been developed, especially from the genus *Streptomyces* due to its ability to produce nematocidal metabolites (Samac and Kindel, 2001; El-Nagdi and Youssef, 2004). For example, *Streptomyces avermitilis*, which produces the fermentation metabolite abamectin, showed nematocidal activity via seed soaking treatment of fava beans infested with *M. incognita* (El-Nagdi and Youssef, 2004). Although the application of these biocontrol agents can be an environmentally friendly and economical way to overcome disease, the direct application of these strains to soil may result in poor microbiological activity and serious problems because of their impact on non-target organisms, and, more generally, to the biocenosis. In addition, banana growth lasts almost 12 months, and the successful colonization and the establishment of beneficial antagonists are essential for the biocontrol of diseases.

As an environmentally benign agent, endophytes are ideal candidates. Recent reports have observed that endophytes efficiently promoted plant growth and that the endophytes may be biocontrol candidates against plant parasitic nematode (Bogner et al., 2016). An endophytic *Streptomycetes* with siderophore-producing isolated from banana roots was found effective against the Fusarium wilt pathogen (Cao et al., 2005). Previous reports have also observed different kinds of endophytes within various banana tissues (Xia et al., 2011). There are different community structures of endophytes for the different disease levels of banana roots. Investigating the distribution of culturable endophytes in roots and effectively screen for endophytes could improve our knowledge of the antagonistic ability against root-knot nematodes at different disease degrees of banana roots.

Previous researchers have found mixed effects of endophyte presence on soil microbial parameters (Iqbal et al., 2012) and reductions in root nematode herbivory (Timper, 2009). For example, some studies have demonstrated that exudates from endophyte infected host could stimulate soil microbial activity (Hecke et al., 2005) which in turn can lead to variation in the nematode community (Yang et al., 2015). Nematode community structure analysis has been used to assess environmental quality (Bongers, 1990; Yeates, 2007). Nematodes are used as appropriate indicators of soil ecosystem resilience due to the presence of multiple feeding groups participating in soil food webs (Bongers and Bongers, 1998), which provides insight into ecosystem resilience where larger and more diverse assemblages reflect a capacity to perform numerous ecological functions and therefore sustain soil productivity and health (Yeates, 2007). Variations of the soil nematode community after the introduction of functional endophytes should warrant the same contemplation and research.

To our knowledge, no reports have yet described the endophyte community structure of different disease levels of banana roots or focused on the biocontrol ability of these powerful candidates against root-knot nematodes. Endophytes in banana root more likely infected great numbers of Cavendish varieties on sandy loam soils under tropical conditions. The aim of this study was conducted to (1) compare the abundance of different culturable endophytes with biocontrol ability against plant-parasitic nematodes in three different disease levels of banana root in Hainan province, China; (2) isolate highly effective endophytic strain for suppressing plant-parasitic nematodes; and (3) investigate its subsequent effect on nematode community composition.

## Materials and Methods

### Root and Soil Collection

The root and soil samples were collected from a field with banana monoculture for 12 years at the WanZhong banana orchard in Hainan, China, and a serious infestation of root-knot nematodes (over 200 individuals per 100 g dry weight soil) have occurred. Mean precipitation measurements and annual temperature in this area were approximately 1150 mm and 24°C, respectively. The soil was loam sandy (sand:silt:clay = 46:38:16) and has pH, organic matter, total nitrogen, nitrate nitrogen (NO_3_^-^-N), ammonium nitrogen (NH_4_^+^-N), available potassium, and available phosphorus contents of 5.6, 7.6 g kg^-1^, 0.4 g kg^-1^, 143.9 mg kg^-1^, 10.3 mg kg^-1^, 278.2 mg kg^-1^, and 173.6 mg kg^-1^, respectively. Triplicate root samples were collected and homogenized separately and classified into three disease levels (i.e., low, middle, and high), according to banana plants infected with galls. All roots without obvious galls were classified into low disease level (low); half of roots infected with galls were classified into middle disease level (middle); and all roots infected with galls were classified into high disease level (high). All samples were transferred to library at room temperature and processed in sampling day. Some of the soil samples were sterilized at 121°C for 3 h for later use if necessary.

### Isolation of Endophytes from Root Samples

The root surface was brushed using sterilized water first, and then sterilized according to Cao et al. (2004) with some modifications. In brief, a triplicate of each fresh root sample (5 g) were placed in a 5% sodium hypochlorite solution (100 ml) and shaken for 5 min, followed by immersion in 70% ethanol for 10 min to ensure complete surface-sterilization. Later, the root surface was washed three times using sterilized water to remove sterilization agents. For the endophyte counts, surface-sterilized roots were cut into 1-cm sections, macerated with sterile 0.85% NaCl solution (5 ml) independently, and homogenized by a mortar and pestle. The culturable microbes were counted using a standard 10-fold dilution method. In brief, dilute root suspensions were inoculated onto plates containing R2A medium (Reasoner and Geldreich, 1985) for bacteria and Martin’s Rose Bengal agar medium (Shen et al., 2013) for fungi, respectively. In parallel, surface sterilization was confirmed using the same method and media for three times. Bacteria and fungi were incubated at 30°C for 4–6 days and 28°C for 3 days, respectively, and then observed using a microscope (Shen et al., 2013). The results were expressed as CFU per gram of root. The colonies with different morphological characteristics and odor properties from the plates of each sample were preliminarily chosen for different strains. Similar colonies were enumerated, and one was selected. A total of 100 bacterial isolates and 10 fungal isolates were obtained from each treatment and purified before storage at -70°C for subsequent DNA extraction.

### Cultures of Nematodes and Antagonistic Tests *in Vitro*

The egg masses of *Meloidogyne javanica* were collected according to Yang et al. (2015) and cultured on banana seedlings (*Musa acuminate* AAA *Cavendish*) in sterilized soil in the greenhouse. After 60 days, egg masses were collected from the roots with deionized water. Second-stage juveniles (J2s) were obtained from all egg masses which were placed in plates with 5 ml water and hatched at 25°C for 72 h (Yang et al., 2015).

For the detection of inhibiting activity, the bacterial isolates were cultured in liquid LB medium at 30°C (180 rpm) for 36 h and the fungal isolates were cultured in liquid potato dextrose medium (PDA) for 96 h at 28°C. The fermentation broth was centrifuged and 200 μl of supernatant was transferred to a Petri dish (5.5 cm diameter) containing 200 individual freshly hatched J2s of *M. javanica*. A dish amended with an equal volume of liquid medium was performed as a control. Totally, 110 isolates and control were performed in triplicate. The dishes were incubated at 25°C for 48 h, then Olympus ZX10 stereomicroscope was used to observe the nematodes and the corrected inhibiting rate of activity was calculated. Nematodes were considered paralytic if they did not move when probed with a fine needle and to be active if they moved or appeared as a winding shape. Then, the nematodes in each treatment were transferred to distilled water for 48 h to ascertain whether the paralytic nematodes regained mobility or not (Yang et al., 2015). The corrected inhibiting rate of activity was calculated according to the following formula: inhibiting rate (%)=(inhibiting rate of treatment-the inhibiting rate of control)/(1-the inhibiting rate of control)× 100. This experiment was repeated twice. In total, 36 bacterial strains with corrected inhibiting rate over 50% were chosen for subsequent characterization, root colonization, and biocontrol efficiency determination.

### Characterization of Selected Strains

The 36 isolated bacterial strains were identified according to Li et al. (2011, 2014). Genomic DNA was extracted, and the 16S rRNA gene was amplified using polymerase chain reaction (PCR) using the following primers: 5′-AGA GTT TGA TCC TGG CTC AG-3′ (forward) and 5′-TAC GGT TAC CTT GTT ACG ACT T-3′ (reverse). The 16S rRNA gene sequence of all strains was sequenced and analyzed using BLAST searches. The phylogenetic tree was built with the neighbor-joining method.

### Determination of Root Colonization and Biocontrol Efficiency of Endophytes

The growing points of banana bulbs were surface-sterilized and inoculated into sterile tissue culture flasks with agar medium [1/2 MS (Murashige and Skoog, 1962), NaCl 10 g, sucrose 30 g, agar 8 g, active carbon 1 g, distilled water 1000 ml, pH 6.0]. Sterile banana seedlings, which were cultured in a sterilized seedling matrix according to Yang et al. (2015), were arranged in a sterile culture chamber. A 100-μl fermentation medium with approximately 10^8^ CFU/ml of each strain was amended in the banana rhizosphere when the banana seedlings had four leaves and 8 cm height. The plants were maintained in a sterile culture chamber for 60 days at 26 ± 3°C for 16 h/day of supplemental artificial light/day for hardening. Plants treated with liquid medium were performed as controls. After 60 days, the roots of each banana seedling were weighed and surface-sterilized and strains colonized in roots were detected as described in the previous paragraph. Data were expressed as CFU per gram of root (Brimecombe et al., 2001). Experiments were repeated twice, and each treatment contained six plants.

Antagonistic effects of 36 bacterial isolates were tested against *M. javanica*. Banana seedlings colonized with endophytes were transplanted in plastic pots (15 cm diameter) containing sterilized soil substrate (sand:loam:organic manure = 49:49:2, v/v/v) which were sifted through a 2-mm mesh sieve and sterilized at 121°C for 2 h. Plantlets were acclimatized in a sterile culture chamber at 28 ± 1°C with 16 h of light/day and 60–70% humidity for 2 weeks. Later, 3 ml of nematode suspension (200 J2s/ml) was uniformly added into five holes in the soil substrate for each treatment; 2 months after inoculation, the plants were harvested, and the number of nematodes in roots was counted. Each treatment was set up as a randomized complete block and replicated six times. The experiment was repeated twice. The biocontrol efficiency percentage was calculated as follows:

(1) Biocontrol⁢ efficiency[%]=(m−n)/m×100

where m and n indicate nematodes in the roots of the control and treatments, respectively.

The strains that colonized the root and had biocontrol efficiency over 50% in both tests were chosen for the subsequent experiments.

### Greenhouse Experiments

Considering the short biological cycle of *Meloidogyne* spp., two greenhouse experiments were conducted to evaluate the biocontrol efficiency of selected bacterial strains in diseased soil from April to October 2015 in a greenhouse at the WanZhong Agricultural Company. The soil was collected from the banana field which was infected with approximately 600 plant-parasitic nematodes (over 150 individuals of *Meloidogyne*) per 100 g dry weight soil. The soil was sandy and had a pH of 5.4, total nitrogen content of 0.4 g kg^-1^, NH_4_^+^-N content of 10.2 mg kg^-1^, NO_3_^-^-N content of 141.2 mg kg^-1^, organic matter content of 7.2 g kg^-1^, and available P and K contents of 171.2 mg kg^-1^ and 270.1 mg kg^-1^, respectively. Four treatments consisting of germ-free banana seedlings colonized by four endophytes [named BA (for *Citrobacter freundii* strain BRN1), BB (for *Pseudomonas* sp. JQ2-6), BC (for *Pseudomonas nitroreducens* strain TX1), and SA (for *Streptomyces capoamus* strain p14 C01)] for 30 days, as described in the previous paragraph, and a control (CK) without the endophyte were designed with three replicates containing nine pots for each one. All treatments were amended with the same amount of pig manure compost (30 g kg^-1^ soil, dry weight). Before each greenhouse experiment, all required fertilizers were amended to the soil once. After the first season greenhouse experiment, the plants were removed, and the soil containing over 300 individual nematodes (over 100 plant-parasites) per 100 g dry weight soil in each pot was left fallow. For the second greenhouse experiment, all treatments were amended with the same amount of pig manure compost (30 g kg^-1^ soil, dry weight) as described previously. Banana tissue culture seedling was planted in each pot in order to simulate a continuous cropping to detect whether the application of banana seedlings colonized antagonistic endophytes could still act as an efficient biological control method for plant-parasites.

At the end of the greenhouse experiment (planting for 80 days), three plants were randomly selected for each replicate and combined to form a subsample. Three subsamples were obtained for each treatment. Root samples were collected according to Su et al. (2015) and plant-parasites in roots were extracted and calculated following Yang et al. (2015). The soil next to the banana roots from three pots was randomly chosen from each replicate and combined to form a subsample. Three subsamples were obtained for each treatment and chosen for nematode community analysis.

### Nematode Community Diversity and Ecological Indices Analysis

The total number of nematodes in soil samples were extracted by the Baermann funnel method and counted using an Olympus ZX10 stereo microscope (Yang et al., 2015). Nematodes were assigned to functional guilds according to trophic groups (Djigal et al., 2012) and colonizer–persister classes (Bongers and Bongers, 1998). The maturity index (MI) was calculated as the weighted average of colonizer–persister (c–p) values for each non-plant-feeding taxon according to the 1–5 c–p scale defined by Bongers and Bongers (1998) and Djigal et al. (2012). The plant-parasitic index (PPI) was calculated in the same manner as the MI but using only herbivorous nematode data (plant-feeders and root-hair-feeders) (Djigal et al., 2012). The Shannon index (H′) (Shannon, 1948) was used to calculate the taxonomic diversity of the nematode community. Soil food web indices based on nematode abundance [enrichment index (EI), structure index (SI)] were used to infer soil food web condition (Ferris et al., 2001) along the cropping cycle (Sánchez-Moreno et al., 2010). The diversity and ecological indices were calculated as follows:

(1) Maturity index (Bongers, 1990), MI = ∑ *c* - *pi* × *f(i)*, where *c–pi* is the *c–p* value of free-living (or plant-parasitic) nematode genus *i* according to their r and K characteristics, and *f(i)* is the frequency of genus *i* in the total number of free-living (or plant-parasitic) nematodes in a sample;
(2) Shannon index (Shannon, 1948), H^′^ = -∑ *pi* × ln *pi*, where ‘*pi*’ is the proportion of the individuals in the *i*-th taxon;
(3) Enrichment index (Ferris et al., 2001), EI = 100×e/(e+b), where *e* and *b* were the abundances of individuals in guilds representing enrichment (*e*) and basal (*b*) food web components. These components are calculated based on the weighted abundance of guilds Ba_1_ and Fu_2_ (*e*), and Ba_2_ and Fu_2_ (*b*). The EI assesses food web response to the availability of resources.
(4) Structure index (Ferris et al., 2001), SI = 100 × s/(s + b), where *s* and *b* were the abundance of individuals in guilds representing structured (*s*) and basal (*b*) food web components. These components are calculated based on the weighted abundance of guilds Ba_2_ and Fu_2_ (*b*), and Ba_3-5_, Fu_3-5_, Om_4-5_, and Ca_2-5_ (*s*). The SI indicates whether the soil community is basal (typical of disturbed systems) or structured (typical of more stable systems).

### Data Analysis

Normality and homogeneity of all data were tested using Kolmogorov–Smirnov Test and Levene’s Test in IBM SPSS Statistics 19.0 (SPSS, Inc., Chicago, IL, United States), which was also used for all statistical analysis. Differences in the distribution of culturable endophytes against nematodes at different disease levels and four strains of plant-parasites in greenhouse experiments were assessed using *t*-test or a one-way analysis of variance (ANOVA) for the nematode community diversity analysis between SA and control, and the calculated means were subjected to Duncan’s multiple range test at *p* < 0.05. The Mann–Whitney *U*-test of Non-parametric tests was used to assess differences when the data distribution was skewed.

## Results

### Distributions of Culturable Endophytes

**FIGURE 1:**
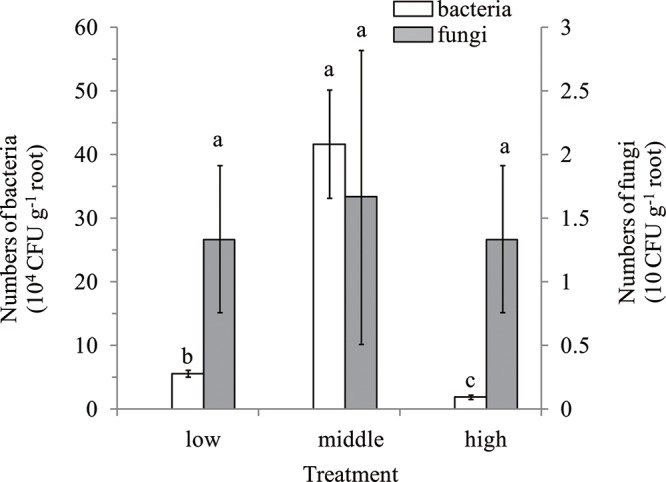
Enumeration of cultivable endophytes in roots of different disease levels. Means were calculated from three replications. Bars with different small letters (a,b,c) indicate significant differences among different disease levels of banana roots as defined by Duncan’s test (*p* < 0.05); low, roots of low disease level; middle, middle disease level; high, high disease level. The *Y*-axis on left was for bacteria, and for fungi on the right.

There was no colony observed on the R2A and Martin’s Rose Bengal agar media, which were plated with flow-out from the washings of surface-sterilized root samples, demonstrating that surface sterilization was successful. Culturable bacteria numbers in the banana roots of low, middle, and high disease levels were 5.57, 41.68, and 1.85 (×10^4^ CFU g^-1^ root), respectively, which were all higher than the fungal counts regardless of disease levels (**Figure 1**). Further, there was no significant difference for culturable fungi among the different disease levels.

### Proportion of Endophytes with Antagonistic Ability

**FIGURE 2:**
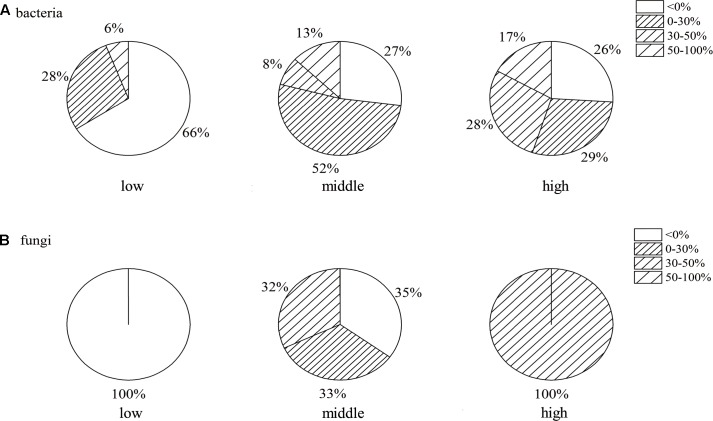
The proportion of antagonistic bacteria **(A)** and fungi **(B)** with corrected inhibiting rate in banana roots of different disease levels. Means were calculated from three replications: low, roots of low disease level; middle, middle disease level; high, high disease level.

The proportion of antagonistic endophytes of 100 bacterial and 10 fungal isolates from the banana roots of three different disease levels is shown in **Figure 2**. Compared to the other two treatments, significantly lower numbers of antagonistic endophytes were observed in the roots of the low disease level, especially for fungi (**Figure 2B**), and no antagonistic endophyte in the roots of low disease level was observed. More than 70% of antagonistic bacteria (**Figure 2A**) and fungi were observed in the roots of middle and high disease levels; higher relative abundance of antagonistic endophytes with an inhibiting rate above 30% was observed in the roots of middle and high disease levels; and 13 and 17% of bacteria with an inhibiting rate of 50–100% were observed in the roots of middle and high disease levels, respectively. No significant difference was observed between the bacteria with the inhibiting rate of 30–50 and 50–100%.

### Root Colonization and Antagonistic Abilities of Endophytes in Soil Substrate

The fungal isolates were not selected for the following tests due to the low biocontrol efficiency (lower than 50%) detected *in vitro*. In total, 36 bacterial isolates with biocontrol efficiency above 50% were chosen for identification, root colonization, and detection of antagonistic abilities. Of the all isolated endophytic species with nematode-antagonistic ability, *Pseudomonas* species showed a maximum relative abundance of 30.6%, followed by *Bacillus* spp. and *Streptomyces* spp., with the relative abundances of 22.2 and 19.4%, respectively (**Table 1**). The relative abundance of other communities, except *Acinetobacter* spp. and *Citrobacter* spp., was evidently lower in roots. A total of 25 bacterial isolates colonized banana roots. The colonization and biocontrol efficiencies of the strains are shown in **Table 1**. There were four strains (BA, BB, BC, and SA) that showed higher antagonistic ability (above 50%) and among those, strain SA belonging to *Streptomyces*, showed the highest biocontrol efficiency of 70.7%. The other three strains (BA, BB, and BC) belonged to *Citrobacter* sp., *Pseudomonas* sp. and *Pseudomonas* sp., respectively.

**Table 1:** Colonization and corrected control efficiency in soil substrate of antagonistic bacteria isolated from banana roots.

Accession	Isolates	Isolates ID	Colonization	Corrected control
			(CFU g^-1^ root)	efficiency (%)
MG004163	*Acinetobacter* sp. MemCl4	10	2.29 × 10^6^	-3.2
MG004161	*Acinetobacter* sp. KL5(2010)	12	2.06 × 10^4^	2.3
MG004167	*Alcaligenes faecalis* strain BAB-1832	6	2.54 × 10^7^	2.1
MG004164	*Bacillus* sp. CH-1	9	1.21 × 10^9^	27.3
MG004162	*Bacillus* sp. BAB-3405	11	4.25 × 10^9^	11.2
MG004155	*Bacillus* sp. 4077	18	0	5.8
MG004154	*Bacillus subtilis* strain S12	19	0	64.1
MG004153	*Bacillus* sp. CZB22	20	0	20.1
MG004152	*Bacillus subtilis* partial	21	0	48.0
MG004151	*Bacillus* sp. SA071_2	22	0	11.2
MG004141	*Bacillus methylotrophicus* strain IS04	32	58	20.1
MG004149	*Cellulosimicrobium* sp. 0707K4-3	24	1.34 × 10^3^	2.0
MG004171	*Chryseobacterium* sp. PNP8	2	3.15 × 10^7^	8.5
MG004143	*Citrobacter braakii* strain A8	30	0	-2.1
MG004140	*Citrobacter freundii* strain BRN1	BA	3.59 × 10^7^	51.0
MG004157	*Enterobacter* sp. KZ_AalM_Mm9	16	0	-3.4
MG004160	*Microbacterium resistens* strain 3352	13	0	20.7
MG004145	*Ochrobactrum* sp. 1605	28	1.76 × 10^4^	-11.2
MG004170	*Pseudomonas* sp. PGB2	3	2.19 × 10^5^	4.1
MG004168	*Pseudomonas nitroreducens*	5	4.03 × 10^3^	38.8
MG004165	*Pseudomonas* sp. 1GW5	8	1.29 × 10^9^	28.6
MG004139	*Pseudomonas* sp. JQ2-6	BB	5.76 × 10^4^	54.5
MG004138	*Pseudomonas nitroreducens* strain TX1	BC	2.41 × 10^5^	59.7
MG004159	*Pseudomonas* sp. JDC-6	14	1.30 × 10^4^	11.2
MG004156	*Pseudomonas* sp. JDC-5	17	1.38 × 10^3^	39.8
MG004169	*Pseudomonas* sp. CAT1-8	4	2.37 × 10^6^	3.9
MG004166	*Pseudomonas* sp. SY66	7	1.04 × 10^4^	3.1
MG004172	*Pseudomonas* sp. Q3	1	3.35 × 10^3^	4.6
MG004158	*Pseudomonas* sp. bD39(2011)	15	2.19 × 10^5^	4.1
MG004150	*Streptomyces cellostaticus*	23	0	-4.8
MG004148	*Streptomyces* sp. An53 partial	25	30	2.7
MG004147	*Streptomyces* sp. An19 partial	26	245	23.6
MG004146	*Streptomyces shenzhenensis* strain 172115	27	515	-6.2
MG004144	*Streptomyces* sp. GXT6	29	0	-2.3
MG004142	*Streptomyces* sp. neau-Q1	31	0	48.8
MG004137	*Streptomyces capoamus* strain p14 C01	SA	205	70.7

*A total of 36 bacterial isolates were analyzed. The letters followed by the species indicated the strains chosen for the following tests*.

### Analysis of Antagonistic Effects against Nematodes in Greenhouse Experiment

**FIGURE 3:**
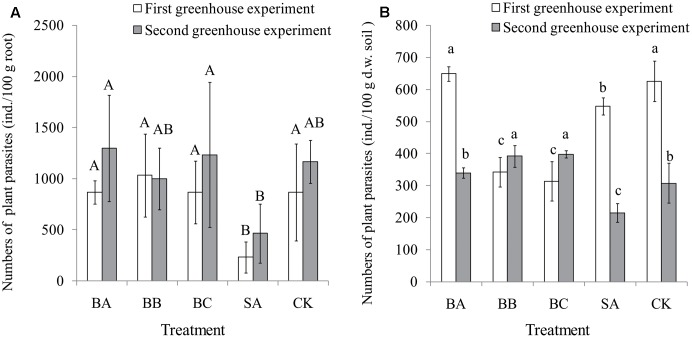
Nematode abundances of root (ind./100 g root) **(A)** and plant-parasites in soil (ind./100 g dry weight soil) **(B)** after harvest (80 days) in the two greenhouse experiments. Means were calculated from three replications. The error line indicates standard deviation of the mean. Bars with different capital letters (A,B) and different small letters (a,b,c) indicate significant differences among the treatments in each greenhouse experiment as defined by Duncan’s test (*p* < 0.05). Control (CK), sterile banana seedlings; BA, banana seedlings colonized with *Citrobacter freundii* strain BRN1; BB, banana seedlings colonized with *Pseudomonas* sp. JQ2-6; BC, banana seedlings colonized with *Pseudomonas nitroreducens* strain TX1; SA, banana seedlings colonized with *Streptomyces capoamus* strain p14 C01.

The results of the two greenhouse experiments showed that SA treatment significantly reduced the abundance of nematodes in the banana roots compared to other treatments and control. The other treatments and control did not show any significant difference among each other, indicating that except strain SA, other treatments were not effective in reducing the nematodes population in the banana roots (**Figure 3A**).

Only the data for plant-parasites in soil are shown here. In the first season greenhouse experiment, significantly fewer plant-parasites were observed in the SA treatment compared to control (**Figure 3B**). In the second season greenhouse experiment, the SA treatment showed the lowest number of plant-parasites compared to other treatments and control, indicating the best antagonistic effect (**Figure 3B**).

### Nematode Trophic Groups, Diversity, and Analysis of Soil Food Web Conditions

Soil samples from SA treatment were selected for subsequent study due to stable antagonistic ability. The results of the two greenhouse experiments showed that, for the tropic groups in the soil, SA treatment led to more bacterivores and fewer plant-parasites compared to control. No significant differences in fungivores, omnivores–predators, or total nematodes were observed in the first season greenhouse experiment, while the number of omnivores–predators in the second season greenhouse experiment was significantly lower in SA treatment (**Table 2**).

**Table 2:** Nematode abundance (ind./100 g dry weight soil) of individual taxons and ecological indices for control and SA treatment after harvest in the two greenhouse experiments.

Nematode taxon	First greenhouse		Second greenhouse	
	experiment		experiment	
	Control	SA	Control	SA
**Bacterivores**				
*Cephalobus*	51 ± 10.80	105 ± 3.63*	57 ± 14.57	122 ± 23.56*
*Prismatolaimus*	16 ± 1.75	15 ± 8.29	6 ± 6.31	2 ± 3.54
*Mesorhabditis*	51 ± 8.70	133 ± 21.96*	11 ± 5.68	183 ± 15.30*
*Geomonhystera*	2 ± 2.96	2 ± 1.72	2 ± 2.80	5 ± 4.89
*Acrobeloides*	8 ± 9.22	6 ± 2.61	2 ± 2.80	0 ± 0.00
Total	127 ± 21.80	261 ± 15.93*	78 ± 5.82	312 ± 37.42*
**Fungivores**				
*Aphelenchoides*	10 ± 5.33	8 ± 6.52	6 ± 6.28	9 ± 2.39
Total	10 ± 5.33	8 ± 6.52	6 ± 7.05	9 ± 2.39
**Plant-parasites**				
*Meloidogyne*	11 ± 3.63	3 ± 3.12*	42 ± 10.93	2 ± 3.54*
*Rotylenchulus*	609 ± 20.22	533 ± 15.07*	250 ± 39.82	209 ± 31.62
*Pratylenchus*	5 ± 5.14	2 ± 1.72	21 ± 14.60	0 ± 0.00
*Tylenchus*	0 ± 0.00	0 ± 0.00	4 ± 4.19	2 ± 2.82
*Ditylenchus*	19 ± 8.66	4 ± 4.74	12 ± 7.96	0 ± 0.00
Total	644 ± 17.34	542 ± 22.34*	329 ± 26.45	213 ± 28.45*
**Omnivores–predators**				
*Aporcelaimus*	14 ± 12.09	19 ± 5.27	100 ± 10.84	33 ± 15.22*
*Mesodorylaimus*	3 ± 2.79	2 ± 1.72	2 ± 2.80	7 ± 4.15
*Mylonchulus*	5 ± 5.14	4 ± 5.04	0 ± 0.00	0 ± 0.00
Total	22 ± 8.25	26 ± 2.52	102 ± 11.73	40 ± 11.84*
Total nematodes	803 ± 20.03	837 ± 25.24	515 ± 35.12	574 ± 35.12
MI	2.05 ± 0.10	1.80 ± 0.10*	3.59 ± 0.13	1.83 ± 0.07*
PPI	2.97 ± 0.01	2.99 ± 0.01	2.95 ± 0.04	2.99 ± 0.01
EI	71.25 ± 5.86	79.54 ± 3.62	38.46 ± 14.34	84.32 ± 1.85*
SI	58.11 ± 12.08	52.11 ± 2.62	96.62 ± 0.12	90.41 ± 2.51
H′	1.02 ± 0.17	1.18 ± 0.06	1.58 ± 0.22	1.42 ± 0.07

*Values are represented as means ± SDs. ^∗^Indicates means of treatment showing significant differences when analyzed by *t*-test and Mann–Whitney *U*-test (*p* < 0.05). Control, sterile banana seedlings; SA, banana seedlings colonized with endophytes (*Streptomyces capoamus* strain p14 C01)*.

Among bacterivores, *Cephalobus* and *Mesorhabditis* were significantly higher in the SA treatment compared to control in the two greenhouse experiments. No significant differences in the numbers of *Prismatolaimus*, *Geomonhystera*, and *Acrobeloides* were observed between the treatment and control. For fungivores, no significant difference was observed in the number of *Aphelenchoides*. Among plant-parasites in the first season greenhouse experiment, higher numbers of *Meloidogyne* and *Rotylenchulus* were present in control. In the second season greenhouse experiment, SA treatment showed significant inhibition of *Meloidogyne* and *Aporcelaimus*. Among the omnivores–predators in the first season greenhouse experiment, no significant difference was observed between treatments.

The results of the two greenhouse experiments showed that MI was significantly lower in SA treatment compared to control, while EI in the second season greenhouse experiment was significantly higher in the SA treatment. No significant differences in the other indexes between SA treatment and control were observed.

## Discussion

The culturable endophytes abundance was investigated in three disease levels of banana roots, and bacteria were found dominant compared to fungi. In the previous reports, endophytic bacteria colonized the intercellular regions in diverse plants with roots, suggesting a major point of entry and niche (Bacon and Hinton, 2006). Higher number of antagonistic endophytes was observed in the roots of middle and high disease levels in our study. We speculated that endophytes with biocontrol ability can be obtained from diseased plant roots, similar to previous findings which have reported that endophytes with nematodes suppression ability can be acquired from infected plants (Xiao et al., 2014). The effect and proportion of antagonistic bacteria in our result were significantly higher than fungi, indicating that bacteria may possess greater potential for the biological control of nematodes. Among the 36 isolated antagonistic endophyte species, *Pseudomonas* spp., *Bacillus* spp., and *Streptomyces* spp. were frequently detected, indicating these kinds of genera were common to be observed in plant tissues (Hallmann et al., 1997; Cao et al., 2003). Most strains can effectively penetrate their host and establish endophytic associations in our result, so they can play a better role in biological control, due to the fact that the strategic application of endophytes before transplantation or pathogen invasion increased biocontrol ability (Bogner et al., 2016). Among all the bacteria, strain SA showed the best antagonistic ability after inner-colonization. In agreement with our results, previous studies have also shown that the endophytic Actinobacteria have been reported for producing bioactive compounds like antibiotics, secreting enzymes to degrade cell wall and competing with pathogens for nutrients (El-Tarabily and Sivasithamparam, 2006). Hence, our present results revealed that *Pseudomonas* spp., *Bacillus* spp., and *Streptomyces* spp. possessed superior inner-colonization and subsequent antagonistic abilities for *M. javanica*, a major nematode pest affecting banana (*Musa* spp.) production in China (Liu et al., 2005), in sterile soil environments.

The two greenhouse experiments showed that among the four strains, only *Streptomyces* sp. SA exhibited excellent antagonistic ability on plant-parasites both in root and in soil. *Streptomyces* against plant-parasites (El-Nagdi and Youssef, 2004) by producing a wide variety of important antibiotics (Samac and Kindel, 2001) is a common actinomycete genus. Avermectin (Burg et al., 1979), nanchangmycin, and milbemycin (Ouyang et al., 1993) isolated from *Streptomyces* sp. are reported to have high antagonistic activity against many harmful plant-parasitic nematodes (Zhang et al., 2016). Moreover, endophytic bacteria that can improve plant establishment under various conditions (Hayakawa, 1990) and promote plant growth (Igarashi et al., 2002) are known to be good candidates for biocontrol of several diseases and pests (Aravind et al., 2010). The co-evolution of endophytic bacteria and plants results in an intimate relationship involving exchange of information at the cellular and molecular levels (Hallmann, 2001; Bacon and Hinton, 2006; Aravind et al., 2010). They colonize the same ecological niches as plant pathogens and therefore may be better suited than rhizosphere bacteria to outcompete or directly antagonize pathogens (Ryan et al., 2008; Aravind et al., 2010). Banana plants derived from tissue culture planting material are free of microorganisms; thus, endophytic actinomycetes can penetrate their sterile plant tissue easily (Cao et al., 2004). If the endophytic actinomycetes were introduced into sterile banana seedlings at the breeding stage, they may become a principal part of the microbial flora of the banana plant at transplantation time and could protect the host plant (Sturz et al., 1999). Utilizing endophytic bacteria for biocontrol purposes would eliminate the need to select bacterial types with an elevated level of rhizosphere competence, which is often considered necessary for successful inoculation on seed or root before or during planting (Sturz et al., 1999; Aravind et al., 2010).

The results showed that SA was an efficient biocontrol endophyte and showed better suppression ability of plant-parasites infection both in roots and in soil than the control after harvest in the two greenhouse experiments. Plant-parasites in roots in the second season greenhouse experiment were obviously increased as compared to the first season greenhouse experiment, while a decrease was observed in soil. The continuous cropping might increase the infection of plant-parasites on banana roots. Dominate genera, *Rotylenchulus*, in the soil of SA treatment was significantly lower than that in the control in first season greenhouse experiment, while no significant difference was observed in the second season greenhouse experiment, indicating that SA showed a variable inhibitory effect on *Rotylenchulus* in soil. Even though the density of *Meloidogyne* in the two greenhouse experiments was low, SA still showed significant suppression compared to control. SA treatment showed higher abundance of bacterivores indicating an increase of bacterial activity and biomass and a faster turnover of nutrients in the soil (Ferris et al., 1998; DuPont et al., 2009). Increased abundances of *Cephalobus* and *Mesorhabditis* were observed in SA treatment both in the two greenhouse experiments. Bacterial feeders with c–p1 (*Mesorhabditis*) may be more sensitive in perturbed soils (Sánchez-Moreno et al., 2010). Although some omnivores–predators (*Aporcelaimus*) were more abundant in the non-treated soil in the second season greenhouse experiment, omnivores–predators, especially sensitive to soil perturbation (Ferris and Ferris, 1974; Wasilewska, 1979), did only show a moderate response to SA treatment. Maturity indices (MI) were greater in the control soil, reflecting the increasing contribution to the nematode fauna by larger, omnivorous nematodes with greater *c*–*p* values (Yeates and Bongers, 1999), suggesting that the food web was disturbed in SA (Bongers, 1990). EI was generally higher in SA treatment in the second season greenhouse experiment, indicating the effect of soil nutrient enrichment of SA would take a long time. SA had negligible influences on ecological indices of the soil nematode community, including H′, PPI, and SI. This might be due to the single soil nematode community and the short length of exposure to SA for our greenhouse experiment (nearly 3 months).

## Conclusion

The results reported here show that bacteria were dominant in three diseased levels of banana roots, showing enormous potential for the biological control of nematodes. A strain SA identified as *Streptomyces* sp., with definite colonization ability inside banana roots, can act as a potential biological control for plant-parasites, especially *M. javanica*, in roots and sterile or diseased soil. All the results indicated that biological control strategy explored in our result is an effective alternative compared to pesticides which may destroy the ecosystem balance and cause harm to people, and antagonistic endophyte *Streptomyces* sp. SA is a promising prospect for control plant-parasites.

## Author Contributions

RL, YR, and QS conceived and designed the experiments. LS, ZS, CT, and YC performed the experiments. LS and RL analyzed the data. RL and QS contributed reagents, materials, and analysis tools. LS and RL wrote the main manuscript text and prepared figures. All authors reviewed the manuscript.

## Conflict of Interest Statement

The authors declare that the research was conducted in the absence of any commercial or financial relationships that could be construed as a potential conflict of interest.

## References

[B1] Aravind R. Eapen S. J. Kumar A. Dinu A. Ramana K. V. (2010). Screening of endophytic bacteria and evaluation of selected isolates for suppression of burrowing nematode (*Radopholus similis* Thorne) using three varieties of black pepper (*Piper nigrum* L.). *Crop Prot.* 29 318–324. 10.1016/j.cropro.2009.12.005

[B2] Bacon C. W. Hinton D. M. (2006). “Bacterial endophytes: the endophytic niche, its occupants and its utility,” in *Plant Associated Bacteria*, ed. Gnanamanickam S. S. (New Delhi: Springer), 155–194. 10.1007/978-1-4020-4538-7_5

[B3] Bogner C. W. Kariuki G. M. Elashry A. Sichtermann G. Buch A. K. Mishra B. (2016). Fungal root endophytes of tomato from kenya and their nematode biocontrol potential. *Mycol. Prog.* 15 1–17. 10.1007/s11557-016-1169-9

[B4] Bongers T. (1990). The maturity index: an ecological measure of environmental disturbance based on nematode species composition. *Oecologia* 83 14–19. 10.1007/BF00324627 28313236

[B5] Bongers T. Bongers M. (1998). Functional diversity of nematodes. *Appl. Soil Ecol.* 10 239–251. 10.1016/S0929-1393(98)00123-1

[B6] Brimecombe M. J. Leij F. A. Lynch J. M. (2001). Nematode community structure as a sensitive indicator of microbial perturbations induced by a genetically modified, *Pseudomonas fluorescens* strain. *Biol. FertIL. Soils* 34 270–275. 10.1007/s003740100412

[B7] Burg R. W. Miller B. M. Baker E. E. Birnbaum J. Currie S. A. Hartman R. (1979). Avermectins, new family of potent anthelmintic agents: producing organism and fermentation. *Antimicrob. Agents Chemother.* 15 361–367. 10.1128/AAC.15.3.361 464561 PMC352666

[B8] Caboni P. Aissani N. Demurtas M. Ntalli N. Onnis V. (2016). Nematicidal activity of acetophenones and chalcones against *Meloidogyne incognita*, and structure–activity considerations. *Pest Manag. Sci.* 72 125–130. 10.1002/ps.3978 25641877

[B9] Cao L. X. Qiu Z. Q. Dai X. Tan H. M. Lin Y. C. Zhou S. N. (2004). Isolation of endophytic actinomycetes from roots and leaves of banana (Musa Acuminata) plants and their activities against *Fusarium oxysporum* f. sp. *cubense.* *World. J. Microbiol. Biotechnol.* 20 501–504. 10.1023/B:WIBI.0000040406.30495.48

[B10] Cao L. X. Qiu Z. Q. You J. L. Tan H. M. Zhou S. N. (2005). Isolation and characterization of endophytic streptomycete antagonists of Fusarium wilt pathogen from surface-sterilized banana roots. *FEMS Microbiol. Lett.* 247 147–152. 10.1016/j.femsle.2005.05.006 15935565

[B11] Cao L. X. Tian X. L. Zhou S. N. (2003). Isolation of endophytic fungi and actinomycetes from banana (*Musa paradisiaca*) plants. *Acta Sci. Nat. Univ. Sunyatseni* 42 70–73.

[B12] Compant S. Duffy B. Nowak J. Clément C. Barka E. A. (2005). Use of plant growth-promoting bacteria for biocontrol of plant diseases: principles, mechanisms of action, and future prospects. *Appl. Environ. Microbiol.* 71 4951–4959. 10.1128/AEM.71.9.4951-4959.2005 16151072 PMC1214602

[B13] Djigal D. Chabrier C. Duyck P. F. Achard R. Quénéhervé P. Tixier P. (2012). Cover crops alter the soil nematode food web in banana agroecosystems. *Soil Biol. Biochem.* 48 142–150. 10.1016/j.soilbio.2012.01.026

[B14] DuPont S. T. Ferris H. Van Horn M. (2009). Effects of cover crop quality and quantity on nematode-based soil food webs and nutrient cycling. *Appl. Soil Ecol.* 41 157–167. 10.1016/j.apsoil.2008.10.004

[B15] El-Nagdi W. M. A. Youssef M. M. A. (2004). Soaking faba bean seed in some bio agents as prophylactic treatment for controlling *Meloidogyne incognita* root knot nematode infection. *J. Pest Sci.* 77 75–78. 10.1007/s10340-003-0029-y

[B16] El-Tarabily K. A. Sivasithamparam K. (2006). Non-streptomycete actinomycetes as biocontrol agents of soil-borne fungal plant pathogens and as plant growth promoters. *Soil Biol. Biochem.* 38 1505–1520. 10.1016/j.soilbio.2005.12.017

[B17] Ferris H. Bongers T. de Goede R. G. M. (2001). A framework for soil food web diagnostics: extension of the nematode faunal analysis concept. *Appl. Soil Ecol.* 18 13–29. 10.1016/S0929-1393(01)00152-4

[B18] Ferris H. Venette R. C. Van Der Meulen H. R. Lau S. S. (1998). Nitrogen mineralization by bacterial-feeding nematodes: verification and measurement. *Plant Soil* 203 159–171. 10.1023/A:1004318318307

[B19] Ferris V. R. Ferris J. M. (1974). Inter-relationships between nematode and plant communities in agricultural ecosystems. *Agroecosystems* 1 275–299. 10.1016/0304-3746(74)90039-0

[B20] Hallmann J. (2001). “Plant interactions with endophtyic bacteria,” in *Biotic Interaction in Plant–Pathogen Associations*, eds Jeger M. J. Spence N. J. (Wallingford: CAB International), 87–120. 10.1079/9780851995120.0087

[B21] Hallmann Q. A. Hallmann J. Kloepper J. W. (1997). Bacterial endophytes in cotton: location and interaction with other plant associated bacteria. *Can. J. Microbiol.* 43 254–259. 10.1139/m97-035

[B22] Hayakawa M. (1990). Selective isolation methods and distribution of soil actinomycetes. *Actinomycetologica* 4 103–112. 10.3209/saj.4_103

[B23] Hecke M. M. V. Treonis A. M. Kaufman J. R. (2005). How does the fungal endophyte *Neotyphodium coenophialum* affect tall fescue (*Festuca arundinacea*) rhizodeposition and soil microorganisms? *Plant Soil* 275 101–109. 10.1007/s11104-005-0380-2

[B24] Igarashi Y. Iida T. Yoshida R. Furumai T. (2002). Pteridic acids A and B, novel plant growth promoters with auxin-like activity from *Streptomyces hygroscopicus* TP-A0451. *J. Antibiot.* 55 764–767. 10.7164/antibiotics.55.764 12374388

[B25] Iqbal J. Siegrist J. A. Nelson J. A. Mcculley R. L. (2012). Fungal endophyte infection increases carbon sequestration potential of southeastern United States tall fescue stands. *Soil Biol. Biochem.* 44 81–92. 10.1016/j.soilbio.2011.09.010

[B26] Lamovšek J. Urek G. Trdan S. (2013). Biological control of root-knot nematodes (Meloidogyne spp.): Microbes against the pests. *Acta Agric. Slov.* 101 263–275. 10.2478/acas-2013-0022

[B27] Li R. Li L. Huang R. Sun Y. Mei X. Shen B. (2014). Variations of culturable thermophilic microbe numbers and bacterial communities during the thermophilic phase of composting. *World J. Microbiol. Biotechnol.* 30 1737–1746. 10.1007/s11274-013-1593-9 24415499

[B28] Li R. Zheng J. W. Ni B. Chen K. Yang X. J. Li S.-P. (2011). Biodegradation of pentachloronitrobenzene by *Labrys portucalensis* pcnb-21 isolated from polluted soil. *Pedosphere* 21 31–36. 10.1016/S1002-0160(10)60076-8

[B29] Liu Z. Qin B. Chen Y. Lu X. (2005). Advances in banana root knot nematode in China. *Plant. Prot.* 31 19–21.

[B30] Mendoza A. R. Sikora R. A. (2009). Biological control of *Radopholus similis* in banana by combined application of the mutualistic endophyte *Fusarium oxysporum* strain 162 the egg Pathogen *Paecilomyces lilacinus* strain 251 and the antagonistic bacteria *Bacillus firmus*. *Biocontrol* 54 263–272. 10.1007/s10526-008-9181-x

[B31] Murashige T. Skoog F. (1962). A revised medium for rapid growth and bioassays with tobacco tissue cultures. *Physiol. Plant.* 15 473–497. 10.1111/j.1399-3054.1962.tb08052.x

[B32] Oka Y. Kohai H. Bar-Eyal M. Mor M. Sharon E. Spiegel Y. (2000). New strategies for the control of plant-parasitic nematodes. *Pest Manag. Sci.* 56 983–988. 10.1002/1526-4998(200011)56:11<983::AID-PS233>3.0.CO;2-X

[B33] Ouyang L. Tu G. Gao Y. Zhang P. Xie X. (1993). Two insecticidal antibiotics produced by *Streptomyces nanchangensis*. *J. Jiangxi Agric. Univ.* 15 148–153.

[B34] Qin S. Xing K. Jiang J. H. Xu L. H. Li W. J. (2011). Biodiversity, bioactive natural products and biotechnological potential of plant-associated endophytic actinobacteria. *Appl. Microbiol. Bioethanol.* 89 457–473. 10.1007/s00253-010-2923-6 20941490

[B35] Quénéhervé P. (2009). “Integrated management of banana nematodes,” in *Integrated Management of Fruit Crops and Forest Nematodes*, eds Ciancio A. Mukerji K. G. (Dordrecht: Springer), 3–61. 10.1007/978-1-4020-9858-1_1

[B36] Reasoner D. J. Geldreich E. E. (1985). A new medium for the enumeration and subculture of bacteria from potable water. *Appl. Environ. Microbiol.* 49 1–7. 3883894 10.1128/aem.49.1.1-7.1985 PMC238333

[B37] Ryan R. P. Germaine K. Franks A. Ryan D. J. Dowling D. N. (2008). Bacterial endophytes: recent developments and applications. *FEMS Microbiol. Lett.* 278 1–9. 10.1111/j.1574-6968.2007.00918.x 18034833

[B38] Samac D. A. Kindel L. L. (2001). Suppression of the root-lesion nematode (*Pratylenchus penetrans*) in alfalfa (*Medicago sativa*) by *Streptomyces* spp. *Plant Soil* 235 35–44. 10.1023/A:1011820002779

[B39] Sánchez-Moreno S. Jiménez L. Alonso-Prados J. L. García-Baudín J. M. (2010). Nematodes as indicators of fumigant effects on soil food webs in strawberry crops in Southern Spain. *Ecol. Indic.* 10 148–156. 10.1016/j.ecolind.2009.04.010

[B40] Shannon C. E. (1948). A mathematical theory of communication. *Bell Syst. Techn. J.* 27 379–423. 10.1002/j.1538-7305.1948.tb01338.x

[B41] Shen Z. Z. Zhong S. T. Wang Y. G. Wang B. B. Mei X. L. Li R. (2013). Induced soil microbial suppression of banana fusarium wilt disease using compost and biofertilizers to improve yield and quality. *Eur. J. Soil Biol.* 57 1–8. 10.1016/j.ejsobi.2013.03.006

[B42] Sturz A. V. Christie B. R. Matheson B. G. Arsenault W. J. Buchanan N. A. (1999). Endophytic bacterial communities in the periderm of potato tubers and their potential to improve resistance to soil-borne pathogens. *Plant. Pathol.* 48 360–369. 10.1046/j.1365-3059.1999.00351.x

[B43] Su L. X. Ruan Y. Z. Yang X. J. Wang K. Li R. Shen Q. R. (2015). Suppression on plant-parasitic nematodes using a soil fumigation strategy based on ammonium bicarbonate and its effects on the nematode community. *Sci. Rep.* 5:17597. 10.1038/srep17597 26621630 PMC4664931

[B44] Timper P. (2009). “Nematodes,” in *Tall Fescue for the Twenty-First Century* Vol. 53 eds Fribourg H. A. Hannaway D. B. West C. P. (Madison, WI: ASA CSSA-SSSA), 151–156. 10.2134/agronmonogr53.c10

[B45] Wang B. B. Yuan J. Zhang J. Shen Z. Z. Zhang M. Li R. (2013). Effects of novel bioorganic fertilizer produced by *Bacillus amyloliquefaciens* W19 on antagonism of *Fusarium* wilt of banana. *Biol. Fert. Soils.* 49 435–446. 10.1007/s00374-012-0739-5

[B46] Wasilewska L. (1979). The structure and function of soil nematode communities in natural ecosystems and agrocenoses. *Pol. Ecol. Stud.* 5 97–145.

[B47] Xia X. Lie T. K. Qian X. Zheng Z. Huang Y. Shen Y. (2011). Species diversity, distribution, and genetic structure of endophytic and epiphytic trichoderma associated with banana roots. *Microb. Ecol.* 61 619–625. 10.1007/s00248-010-9770-y 21063870

[B48] Xiao F. Tang X. C. Wang L. Wu T. (2014). Suppression of *Meloidogyne incognita* by the endophytic fungus *Acremonium implicatum* from tomato root galls. *Int. J. Pest Manage.* 60 239–245. 10.1080/09670874.2014.958604

[B49] Xu L. B. Yang H. Huang B. Z. Wei Y. R. (2004). “Production and R&D of banana in China,” in *Proceedings of the Advancing Banana and Plantain R&D in Asia and the Pacific*, Guangzhou, 49–60.

[B50] Yang X. J. Wang X. Wang K. Su L. X. Li H. M. Li R. (2015). The nematicidal effect of Camellia seed cake on root-knot nematode *Meloidogyne javanica* of banana. *PLOS ONE* 10:e0119700. 10.1371/journal.pone.0119700 25849382 PMC4388532

[B51] Yeates G. W. (2007). Abundance, diversity, and resilience of nematode assemblages in forest soils. *Can. J. For. Res.* 37 216–225. 10.1139/x06-172

[B52] Yeates G. W. Bongers T. (1999). Nematode diversity in agroecosystems. *Agric. Ecosyst. Environ.* 74 113–135. 10.1016/S0167-8809(99)00033-X

[B53] Zhang J. Wang L. M. Li Y. H. Ding S. L. Yuan H. X. Riley I. T. (2016). Biocontrol of cereal cyst nematode by *Streptomyces anulatus* isolate S07. *Australas. Plant Pathol.* 45 57–64. 10.1007/s13313-015-0385-0

